# Blended learning in a biology classroom: Pre‐pandemic insights for post‐pandemic instructional strategies

**DOI:** 10.1002/2211-5463.13421

**Published:** 2022-05-23

**Authors:** Irtaza Tahir, Victoria Van Mierlo, Victoria Radauskas, Wayne Yeung, Alastair Tracey, Rosa da Silva

**Affiliations:** ^1^ 3710 Faculty of Health Sciences McMaster University Hamilton ON Canada; ^2^ 3710 Department of Biology McMaster University Hamilton ON Canada; ^3^ 3710 Arts & Science Program McMaster University Hamilton ON Canada

**Keywords:** biology, blended learning, flexibility, mixed‐methods research, online learning, undergraduate

## Abstract

It is increasingly important to utilize novel approaches to improve student learning. This has become especially relevant throughout the COVID‐19 pandemic. Previous studies have shown positive outcomes of blended learning on student satisfaction. Yet, there are limited data in the field of biology on how blended learning practices correlate with overall student performance. Moreover, there is a dearth of information on student perceptions about how blended learning has impacted their education. Through this study, we present insights on the impact of blended learning in a first‐year cell and molecular biology course. Using mixed‐methods research, we evaluated the impact of a blended learning course format on student performance in the learning categories of knowledge and understanding, communication and application, and critical thinking and inquiry. Using a pre‐ vs. postintervention analysis, we show that a blended learning course model does not change students’ performance on multiple‐choice and short answer assessments when compared to a nonblended learning course model. Through a qualitative assessment of student perceptions and sentiments, however, the implemented blended learning approach does appear to provide significant perceived benefits, including learner flexibility, consolidation of content, and the opportunity to apply course content to the ‘real world’. While we recognize that our report describes a very specific blended learning model, we believe that our findings are generalizable to similar introductory courses. As such, we are confident that our case study will provide course designers with a useful foundation to build future blended learning courses.

## Graphical abstract

Through this study, we show that when compared to traditional course models, blended learning increases student learning flexibility and provides students with the opportunity to apply course content to the real world, without adversely affecting student performance.

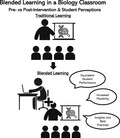

**Abbreviations**
BL: blended learning
CA: communication and application
HOTS: higher order thinking skills
KU: knowledge and understanding
LOTS: lower order thinking skills
TI: thinking and inquiry

The term ‘blended learning’ (BL) has been defined by various educators and researchers since the early 2000s [1, 2, 3]. Shared among many of these definitions is the concept that, at its core, BL is the combination of traditional instructional models and online teaching [1, 3]. Traditional instruction consists of face‐to‐face interactions between students and their instructors (e.g., live lectures, tutorials, etc.). In contrast, online teaching can be inclusive of many forms such as prerecorded modules and lectures, together with other synchronous formats. Allen et al. [1] reference the Sloan Online Learning Consortium, which defines BL as a form of teaching that is a combination of face‐to‐face and online learning, with the online learning component consisting of 30–79% of the entire instructional model. Others argue that BL is a flawed term for what it represents and should instead be termed ‘blended pedagogics’ or ‘blended teaching’ as the key aspect of the model is centered on the method of teaching, rather than how the material is learned [3]. Bonk and Graham [2] define BL as the combination of different teaching styles that are products of different historical origins, which may or may not include electronic or online components.

Although we recognize the varied definitions of BL in the literature, the definition we favor is described by Bliuc et al. [4]: ‘Learning activities that involve a systematic combination of co‐present (face‐to‐face) interactions and technologically mediated interactions between students, teachers, and learning resources’. Many postsecondary educational institutions have adopted BL models for subjects such as science, business, information technology, and nursing [5, 6, 7, 8]. The transition towards BL models has been further embraced by many instructors and educational institutes, given the millions of students at all educational levels who have been displaced from their classroom environments throughout the current COVID‐19 pandemic [9]. Outside of this emergency‐induced movement towards BL, several studies have investigated the impact of BL on student learning performance, together with teacher/faculty reception of BL models. These studies fall under one or more of the following categories; case studies, survey‐type studies, and comparative studies [4]. Overall, findings indicate a positive impression of BL on student satisfaction in multiple disciplines of postsecondary education [8, 10, 11, 12, 13]. Yet, given the heterogeneity between study designs and the various blended learning courses investigated in these studies, these findings are difficult to quantify and compare with new applications of blended learning pedagogy.

## Rationale

As BL becomes increasingly common, questions continue to arise regarding the most effective combination and application of online and traditional teaching practices that can best enhance the learning experience [5, 14]. Investigations of different models of BL are therefore needed to determine whether there is a general optimized model of BL that can be drawn upon and applied by educational institutions in future. Following a review of a vast array of BL‐related studies, Bliuc et al. [4] contend that studies on BL most commonly take the form of case‐study investigations by instructors or institutions providing a BL course. Case studies tend to focus on qualitative aspects of a specific course and aim to improve the course and/or identify its benefits to students in a specific field of study [11, 12]. These case‐study‐based analyses generally conclude that learners feel positively regarding the BL format and perceive BL as providing advantages related to learning and flexibility when compared to traditional classroom models [11, 12, 13].

While case studies do allow for a deep focus into one or a few dimensions of a BL model, the disadvantage to this investigation type is that due to the specificity of the case itself, the qualitative findings of case studies tend to be heavily contextual [4]. As such, deductions from case studies generally become institution‐specific and can make these BL models difficult to implement in other settings. Yet, the disadvantages of case studies can be rectified by complementing the case‐study strategy with other investigative strategies, thereby providing a more holistic analysis of BL implementations [4].

More recently, we have begun to see examples of combined investigative approaches towards evaluating BL interventions. This includes a study carried out at Columbia University, where Stockwell et al. [8] investigated both satisfaction and performance of students enrolled in an undergraduate‐level BL biochemistry course, compared with a traditionally taught version of the same course. As part of their protocol, students were randomized to classes where the same preclass material was delivered as videos (BL) vs. textbook readings (traditional). Student satisfaction and performance were evaluated using qualitative and quantitative measures such as attendance rates, survey questions, and exam scores. It was found that students exposed to the BL formatted course attended class significantly more often than those who attended the biochemistry course that was modeled in a traditional course format. BL students also responded more positively to qualitative questions than those enrolled in the traditionally taught course. Interestingly, when evaluating quantitative exam scores, it was found that these were statistically similar between the BL students and traditionally taught students. Stockwell et al. therefore concluded that while BL may increase student satisfaction, it does not necessarily improve performance. Similarly, Leatherman and Cleveland compared a genetics class that was delivered in a traditional format to the same class delivered in a flipped classroom with online videos delivering course content prior to applied lectures. They too found that there was no difference in student performance between groups. Interestingly, they noted the biggest defining characteristic of students who were dissatisfied with the flipped classroom is their inability to engage with the material in videos [15]. It is important to note that student satisfaction may not be a sole reliable proxy to evaluate the value added by a BL format to a course, making multiple investigative strategies (including both quantitative and qualitative elements) essential when evaluating the outcomes of a BL course on student learning [4, 8, 16].

In 2014, the Department of Biology and McMaster University decided to convert its introductory cellular and molecular biology course Biology 1A03 (BIO1A03) from a traditional classroom to a BL format. This course provides instruction to over 1,600 first‐year undergraduate science students per year, and its conversion to a BL format followed the framework established by the Introductory Psychology Team at McMaster University. The BIO1A03 course redesign involved strategically coordinating instructional design across course components to enhance learning, minimize redundancy, and maximize the application of course concepts to real‐world problems [13]. Within a few years of implementation, the course’s instructional team received a great deal of feedback pertaining to student satisfaction with the course BL format through both informal interactions and the end‐of‐course survey. This resulted in our motivation to design a study where we could engage in an evidence‐based analysis on whether the BL format brought any advantages to students’ learning during the completion of the BIO1A03 course. Specifically, we focused our study on the following overarching questions:

Research Question 1 (RQ1). Is there evidence of changes in student performance after the implementation of blended learning in BIO1A03, both generally and specifically in the areas of knowledge and understanding, communication and application, and critical thinking and inquiry?
Research Question 2 (RQ2). What are students’ perceptions of the blended learning format and its impact on their learning?

Students entering first‐year science programs at McMaster University come primarily from secondary education where they typically undergo a significant degree of traditional in‐person learning. We therefore hypothesized that the novelty of blended learning and the asynchronous components of the course may result in a decrease in assessment performance for students who were enrolled in the postblended learning version of BIO1A03 compared to those who were enrolled in the traditional version of the course. We hypothesized that this general decrease in performance would also be reflected in each of the learning categories. We expected that as students gained familiarity with the blended learning course and developed appropriate learning behaviors, we would see a concomitant increase in student performance as the course progressed. Given the broad variation in student perceptions about blended learning in the literature, we hypothesized that students would have a variation in positive and negative perspectives on blended learning and its impact on their learning.

## Materials and methods

### Study setting and context

This study was conducted using a first‐year cellular and molecular biology course (BIO1A03) at McMaster University (Ontario, Canada). As a large public research university in Canada, the Faculty of Science welcomes approximately 2000 students per year to first‐year studies, with over 1600 of these students enrolling in BIO1A03. BIO1A03 is a four‐month single semester course that is designed to be a student’s first exposure to cell and molecular biology at the undergraduate level and is a prerequisite for most year two programs in the Faculty of Science. Taken together, approximately 700 students are enrolled in the course during each of the fall (September–December) and winter (January–April) semesters, with approximately 200 students enrolled in the spring/summer (May–June/June–August) course offerings. For the purpose of this study, we chose to focus solely on the fall and winter offerings of the course, to ensure consistency between instructors and course duration (fall and winter semesters are 12 weeks in duration, while spring/summer semesters are 6 weeks in duration).

**Fig. 1:**
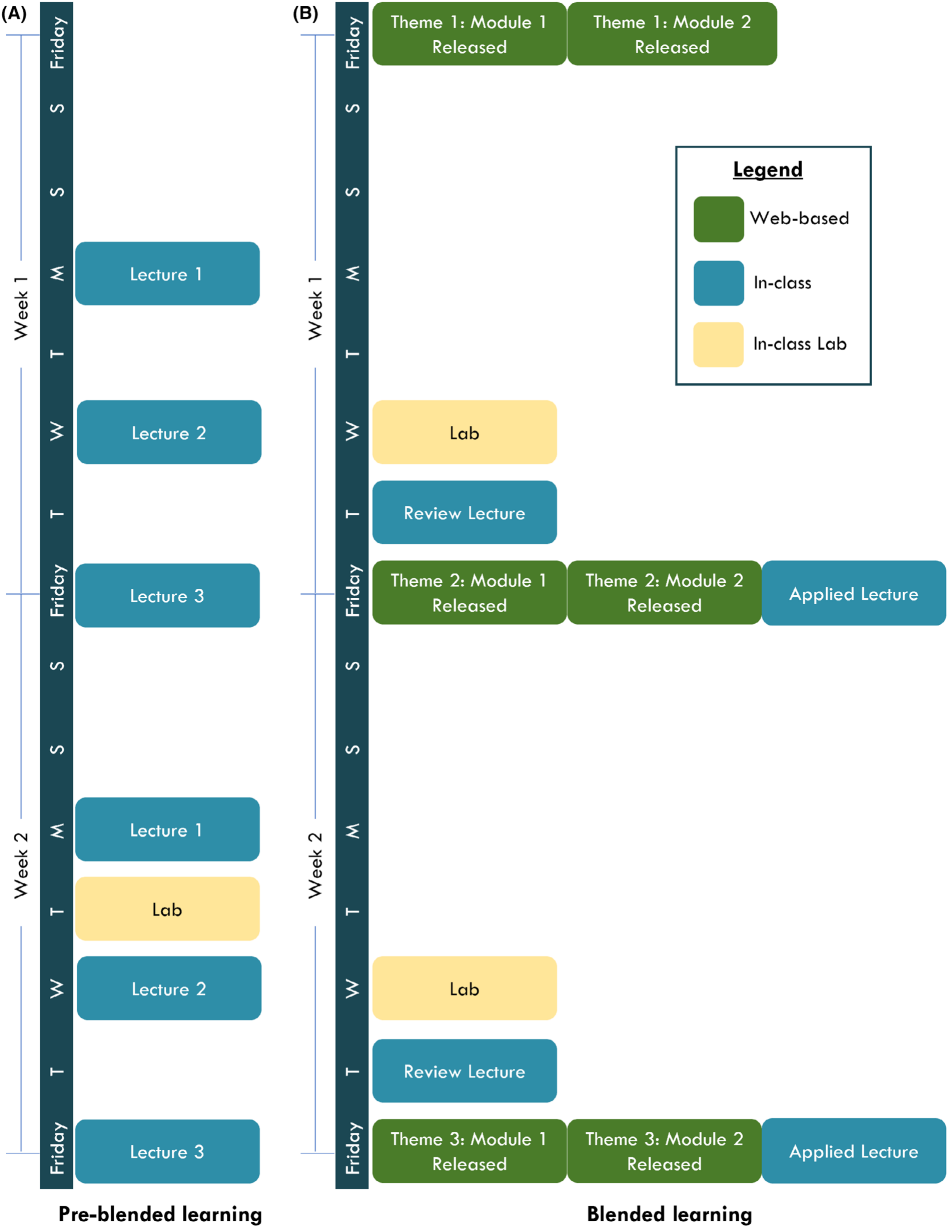
A BIO1A03 course structure comparison. (A) The first 2 weeks of the preblended learning BIO1A03 course. (B) The first 2 weeks of the BIO1A03 course under the blended learning format. These weekly formats continued throughout the semesters.

Prior to 2014, BIO1A03 ran in a traditional, didactic course format, delivered as three 50‐min in‐person (face‐to‐face) lectures per week together with an accompanying three‐hour lab every other week (Fig. 1A). Average lecture section enrollments ranged from 200 to 400 students, with multiple lecture sections running concurrently every semester. The course instructors supplemented students’ learning by using a commercial textbook. Students were evaluated through the completion of two‐term tests (containing multiple‐choice and short answer questions), a multiple‐choice exam, pre‐lab quizzes, lab reports, and a lab exam. All relevant course materials including lecture PowerPoint files and supplementary readings were housed in a learning management system (Desire to Learn, now known as Desire to Learn Brightspace).

During early 2013, the BIO1A03 instructional team was supported by the Office of the Associate Dean of Science to convert BIO1A03 into a BL format course, which was first implemented during the Spring 2014 course offering. The substantial change to a triphasic BL course format (Fig. 1B) included:

1. Independent self‐paced learning that is achieved through prerecorded, in‐house‐built web‐based modules.
2. Face‐to‐face coaching and learning time in the classroom and laboratory.
3. Collaboration and engagement both in the classroom and during weekly laboratories.

With the implementation of BL, the core BIO1A03 course content that was previously delivered within three traditional lectures per week was instead prerecorded and structured into two weekly modules (released every Friday via Desire to Learn) that range in duration from 25 to 40 min. Students are responsible for watching these modules before attending weekly in‐person lectures. In‐person lecture time is divided into two 50‐min lecture blocks every week. The first lecture block, on Thursday, is used as a review lecture where students have the opportunity to work through example questions through student response systems, ask questions related to module content, and instructors are able to provide further clarity on concepts that are difficult for students to understand. Although the review lecture primarily functions to clarify concepts, at times, additional new information and concepts are shared during the review lectures in an attempt to build on existing learning and share practical applications of theoretical course content. The second lecture takes place on Friday, in the format of an ‘applied lecture’. These applied lectures build on the students’ understanding of core course material and are entirely focused on the applications of course concepts to the real world. This often includes in‐depth discussions of relevant real‐world examples, research currently occurring at the university, or science in the news that is directly related to the module content of that week. For example, during the ‘Gene to Protein’ theme of the course, while modules deliver core information related to the processes of transcription and translation, the applied lecture for the week is focused on cystic fibrosis and how we can take advantage of our understanding of the central dogma of biology towards the development of cutting‐edge gene‐editing therapies to treat the disease. Neither review nor applied lectures were recorded or captured for later student use. While the course transitioned to a BL format, in‐person lecture capacity and evaluation methods remained the same.

The lab portion of the course consists of a project‐based lab, which is not aligned with the course content on a week‐per‐week basis but rather encompasses the global broad themes of the course (e.g., variations from gene to protein). In this project‐based lab, students engage in personal genome testing to evaluate variations in gene copy numbers based on ancestry. The evaluation of the project‐based lab and how it aligns with the novel blended learning course model is currently being investigated as a follow‐up paper to this blended learning study.

### Study design

In 2007, Tashakkori & Creswell [17] defined mixed‐methods research as a means of collecting both qualitative and quantitative data in a single study in order to best address a research problem when compared to using either approach on its own. This is important because, while quantitative research methods are able to precisely measure educational phenomena and evaluate the value of educational programs and financial investments, qualitative research methods are essential for capturing contextual information regarding the human and social aspects of education [18]. Due to its holistic, multifaceted, and flexible nature, mixed‐methods research is particularly useful when trying to assess learning in complex social contexts [19], such as in a BL science classroom. Accordingly, over the past several years, there has been an increased appreciation for the need to use mixed methods to understand problems in pedagogy [20, 21]. It is for these reasons that we decided to use a mixed‐methods design for our study as different approaches would be required to answer each of our research questions.

Our study was conducted over two years in two phases. Phase one, focusing on RQ1, occurred over the first year and was suited primarily to quantitative methods. Phase two occurred over the second year and focused on RQ2 using a sequential explanatory design to triangulate a holistic understanding of student perceptions of blended learning [19].

The study was approved by the McMaster University Research Ethics Board (project number 2016 167).

#### Phase 1: Quantitative analysis of student assessment performance

To evaluate the performance of students exposed to a traditional course design, compared with students exposed to the BIO1A03 BL course format, we used a retrospective cohort paradigm, treating blended learning as the exposure of interest.

##### Extraction of quantitative data

Since Fall 2010, the BIO1A03 instructional team has kept records of student marks for each major evaluation (term tests and exams). The anonymization and aggregation of student marks were requisite to our ethics approval to use this pre‐existing data for retrospective analysis. For multiple‐choice questions, the records specify the percent of students that achieved a correct response for each question (marks are not linked to student IDs), while records of short answer questions specify each student’s aggregate mark on each assessment (marks are linked to a student ID). Quantitative data were extracted and anonymized (by team member AT) where necessary, by replacing student IDs with a coded identifier. To develop comparable cohorts, we chose to compare the term test and final exam marks for four‐course offerings prior to the implementation of the BIO1A03 BL course (i.e., fall 2012, winter 2013, fall 2013, winter 2014, which are referred to as preblended learning in this study) to the first four‐course offerings of the BL course format (i.e., fall 2014, winter 2015, fall 2015, winter 2016, which are referred to as postblended learning in this study). Using these course offerings was particularly beneficial, as it was delivered by the same group of instructors with consistency in the length of the exam, the proportion of questions devoted to different content areas, and answer keys (thereby minimizing the impact of additional factors on the between semester variation in student performance).

##### Analysis

Our research team chose to base both the qualitative and quantitative analyses of this study on Bloom’s revised Taxonomy and, specifically, the format adapted by the Ontario Ministry of Education Curriculum K‐12 [22]. According to Bloom et al., learning objectives can be categorized as cognitive, affective, and psychomotor. In the revised taxonomy by Anderson and Krathwohl, the cognitive category of learning objectives is described as consisting of a hierarchy of cognitive functions from lower order thinking skills (LOTS) to higher order thinking skills (HOTS) [23, 24, 25]. The categories are listed from LOTS to HOTS in the following order: (a) remembering and understanding, (b) applying and analysing, and (c) evaluating and creating. Bloom’s taxonomy rests on the notion that in order to achieve any one of the HOTS, one must first achieve all LOTS preceding it. Ultimately, as educators we strive towards pushing students towards higher levels of Bloom’s processing, justifying its use in the assessment of student performance. The Ontario Ministry of Education Curriculum adopts a modification of this revised hierarchy, which also consists of three categories of learning. These categories are referred to as ‘KCAT’ and can be ordered from LOTS to HOTS: knowledge and understanding (KU), communication and application (CA), and thinking and inquiry (TI). Knowledge and understanding incorporates basic comprehension and retention of factual material. Communication and application includes the ability to communicate the material to others and apply the material to situations in a meaningful way. Thinking and inquiry involves the ability to problem solve and ask meaningful questions beyond the material learned. Our rationale to choose the KCAT model in this study was two‐fold. Firstly, there is no established universal postsecondary curriculum framework, thus following a well‐known cognitive domain such as Bloom’s Taxonomy was intuitive and has been implemented in the past [26]. Secondly, most students enrolled in the BIO1A03 course completed high school in Ontario and arrived at McMaster University from the Ontario high school system, and thus were already familiar with the KCAT framework, making it a good proxy for evaluating performance both quantitatively and qualitatively. As a result, questions on tests and exams were categorized as belonging to one of the following categories: ‘knowledge and understanding’, ‘communication and application’, and ‘thinking and inquiry’ as defined by the Ontario Ministry of Education [22]. To reduce subjectivity, questions were categorized independently by two team members with a third acting as a tiebreaker when necessary.

Before we could compare students’ performance on these assessments, we needed to determine whether the structure of these assessments had changed at the same time as the implementation of blended learning, as that would introduce a confounder. To do this, we compared the structure of assessments before and after the implementation of blended learning by calculating the average proportion of questions belonging to each KCAT learning category within the assessments and performing a two‐tailed t‐test assuming unequal variances.

Comparisons between students’ multiple‐choice marks (pre‐ and post‐BL) were determined by calculating and comparing the average proportion of students that achieved a correct response before vs. after BL implementation and were statistically validated using a two‐tailed student’s *t*‐test. Similarly, comparisons between student aggregate short answer marks were determined by calculating and comparing the average mark before vs. after BL implementation and were statistically validated using a two‐tailed *t*‐test assuming unequal variances.

Comparisons between student multiple‐choice marks in each of the categories of KU, CA, and TI were evaluated by isolating the questions for each of those categories, calculating and comparing the average proportion of students that achieved a correct response before vs. after BL implementation for each learning category, with results validated using a two‐tailed t‐test assuming unequal variances. Changes in students’ short answer marks in each learning category could not be extracted as only aggregate marks were available through our records.

To evaluate students’ changes in test performance within a semester under the traditional format compared with the BL format, the difference between term test 1 and term test 2 short answer grades were calculated. This difference in grades provides a quantitative assessment of within‐semester changes in student performance. The within‐semester student performance change was then compared between students who enrolled in the traditional preblended learning course format and those that enrolled in the BL course. Results were validated using a two‐tailed *t*‐test assuming unequal variances. The impact of blended learning on within‐semester changes in students’ multiple‐choice marks could not be calculated as multiple‐choice marks linked to specific student identifiers were not available in our institutional records database. Database organization and analysis were completed using Microsoft Excel v2100 and R v3.1.2, respectively.

#### Phase 2: Quantitative and qualitative analysis of student perceptions

**Fig. 2:**
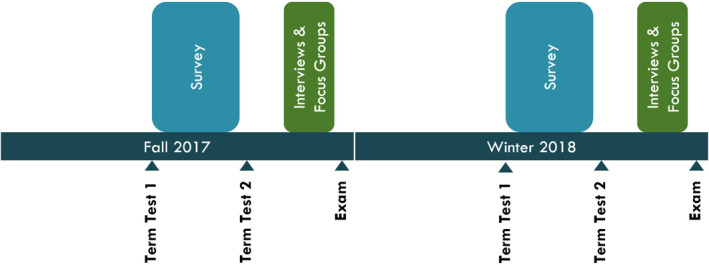
A sequential timeline outlining the phase two design of our study.

According to Krathwohl and Bloom, learning also consists of an affective domain, that is, related to beliefs, attitudes, values, emotions, and acceptance or rejection [27]. Bohlin’s compiled list of components encompassed by the affective domain includes anxiety, arousal, attitude, attributions, beliefs and opinions, confidence, expectancy of success, interests, motivational level, motives, perceived relevance, satisfaction, self‐efficacy, and values [28]. Our second research question, which sought to capture these human and social impacts of the impact of BL on student perceptions, was therefore significantly more complex. Accordingly, we chose to investigate the question using both surveys and focus groups. Our rationale was that surveys were more likely to return responses, allowing us to obtain a large enough sample size to be confident in our conclusions, but that semistructured interviews/focus groups would allow us to dive deeply into students’ sentiments, and correlate them to the survey results. We used a sequential explanatory design consisting of a quantitative cross‐sectional survey, followed by qualitative interviews and focus groups (Fig. 2), to capture students’ perspectives and perceptions of the BL course format and its impact on their learning.

##### Study participants

Students qualifying for participation in the surveys and focus groups included those who were enrolled in the fall 2017 and winter 2018 semesters of BIO1A03. Each semester, students were informed about survey and focus group opportunities both via in‐class and online announcements on Desire to Learn. Students were incentivized to participate in the survey and focus groups by being automatically entered in random draws that provided an opportunity for prizes with study participation. Students were assured that participation or refusal to participate in the study would have no effect on their performance in the course.

##### Quantitative survey

###### Survey design

The survey was constructed and delivered through the LimeSurvey® platform. It consisted of 30 questions covering a broad range of concepts including demographic information, targeted questions pertaining to student perceptions regarding the BL course format, and questions related to their sentiments regarding the impact of blended learning within the three learning categories (i.e., KU, CA, and TI). The survey consisted primarily of Likert‐scale types of questions. These questions were generated by the team to best assess student perceptions of BL in BIO1A03 and elaborate on data that was extracted from Phase 1 of the study. A large portion of these questions was also based on a pre‐existing end‐of‐semester BIO1A03 course survey. Students were notified of their rights as research participants and were asked to provide informed consent at the beginning of the survey. At the end of the survey, students were reminded of the focus groups, and participation was encouraged.

###### Data collection and analysis

Each semester, the survey was released one week after the first term test (around week 8 of the course) and was available to students for two weeks. Survey results were exported from the LimeSurvey platform to Microsoft Excel. Descriptive statistics and graphs were generated using Microsoft Excel.

Survey questions are available in the Supplementary Materials.

##### Qualitative interview and focus groups

###### Interview protocol

The protocol for this semistructured interview was generated by the research team following appropriate McMaster Research Ethics Board Guidelines. Fifteen questions were created specifically to capture information complementary to the survey questions and were standardized across all interviews and focus groups. Although the research team initially intended to conduct focus groups, in most sessions only one student participated per session. Consequently, we adapted the wording of the questions to be appropriate for an interview format as needed. Students were notified of their rights as research participants and were asked to complete informed consent forms before participating in the focus groups, or when needed, in interviews.

###### Data collection and analysis

During each semester, the focus groups were conducted over 10 days, ending 1 week prior to the final exam period. With student consent, the interviews and focus groups were recorded and transcribed verbatim by three members of the team. Transcripts were anonymized and transferred to nvivo 11 pro Qualitative Analysis Software (QSR International, Melbourne, Australia). IT and VV reviewed each transcript and analysed them for recurring themes. We used the six‐phase approach to the qualitative analysis described by Braun and Clarke [29]. As described, we performed a deductive or ‘theoretical’ analysis, focusing less on generating a description of the overall data, and more on detailed coding and analysis to find answers to the question prompts in the focus group guide. We coded primarily at a semantic or explicit level, coding at the surface meanings of the students’ responses. We also coded at a latent level, to interpret underlying ideas that students might have. First, transcripts were read several times for familiarization with, and identification of the data and distinct reoccurring themes. Second, we open‐coded one transcript together (without pre‐emptive bias or categories/framework), to generate the initial coding structure of responses students had regarding the interview questions. Following this, we coded the remaining transcripts independently and then merged our codes. We then sorted related codes into higher level candidate subthemes and themes (axial and selective coding). At times, we used our questions as an *a priori* structure for the codebook. We reviewed these themes and their structure and relationships to ensure that the coded extracts fit that theme. At this stage, we reviewed the transcripts once more to account for any additional data within the themes that may have been missed in earlier coding stages. No new codes emerged by the second‐to‐last interview transcript, indicating data saturation. Representative quotes for each question were then selected by the authors and are presented verbatim with some minor modifications for ease of reading. Finally, survey findings were associated with findings from the interviews.

## Results

### RQ1: Evidence of changes in student performance

**Fig. 3:**
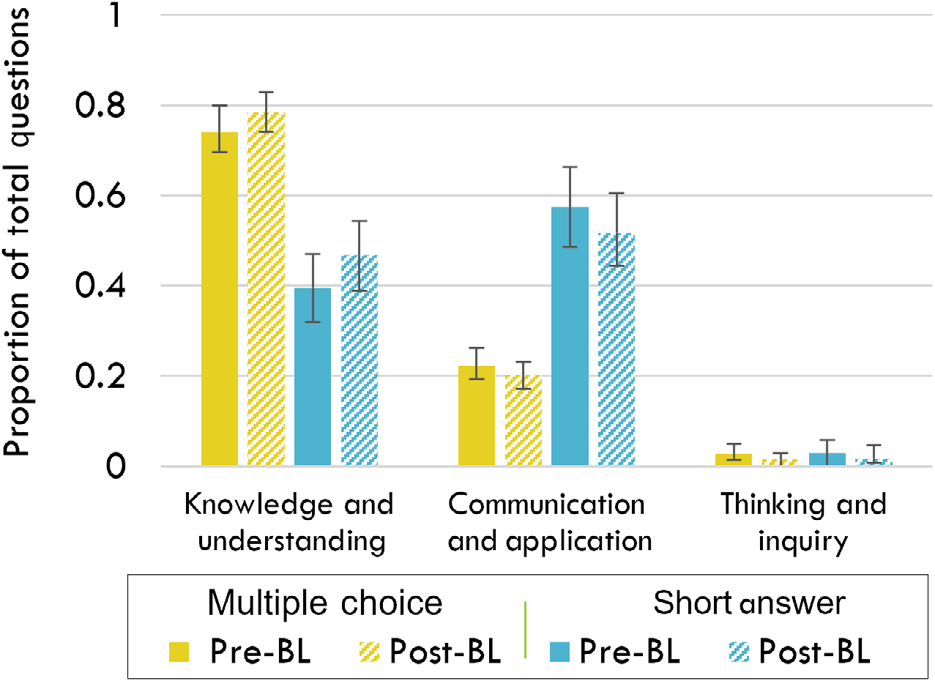
An evaluation of the proportion of questions on tests and exams for each of the three learning categories [knowledge and understanding (KU); communication and application (CA); and thinking and inquiry (TI)] in the BIO1A03 cellular and molecular biology course at McMaster University. The proportion of questions for each learning category was the same in‐course assessments before (Pre‐) and after (Post‐) the implementation of blended learning. Error bars are SEM, *P* > 0.05 (two‐tailed *t*‐test assuming unequal variances); *N* = 4 for each column.

To answer RQ1, we retrospectively compared student performance on major assessments (term tests 1 and 2, and the final exam) before and after the implementation of BL in the BIO1A03 course. To do this, an important first step was to establish that the structure of the assessments (that is, the relative proportions of questions belonging to each learning category‐KU, CA, and TI) had not changed. We found that there was no significant difference in the proportion of each question type in the traditional model of the course when compared to the BL format of the course. This was true for both multiple‐choice and short answer questions (Fig. 3).

**Fig. 4:**
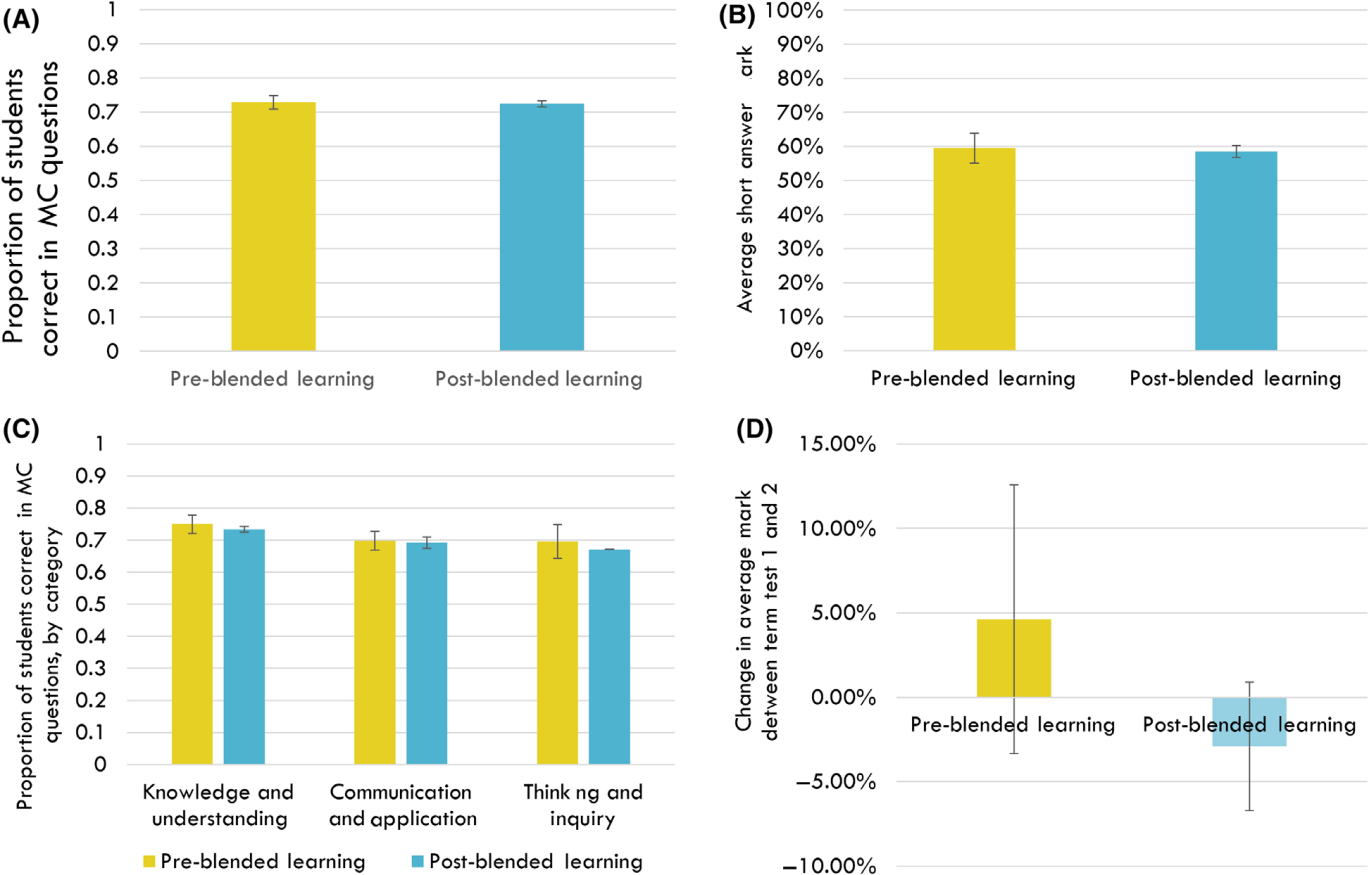
Student performance on assessments in the BIO1A03 cellular and molecular biology course at McMaster University, before (Pre‐) and after (Post‐) the implementation of blended learning (BL). (A) The proportion of students who scored multiple‐choice questions correctly. (B) The average test scores on the short answer components of tests when comparing between before and after the implementation of BL in the course. (C) Multiple‐choice test score within each learning category [knowledge and understanding (KU); communication and application (CA); and thinking and inquiry (TI)], and (D) average within‐semester change in short answer marks between term tests 1 and 2 when comparing between before and after the implementation of BL in the course. In all figures, error bars represent SEM, *P* > 0.05 (two‐tailed *t*‐test assuming unequal variances); *N* = 4 for each column in (A), *N* = 2729 and 2752 students for each respective column in (B), *N* = 4 for each column, except TI postblended learning (*N* = 1) in (C), and *N* = 2729 and 2752 students for each respective column in (D).

When comparing the gross student marks in the BIO1A03 course both before and after the implementation of blended learning, we found no statistically significant difference in the average proportion of students who obtained a correct multiple‐choice answer (Fig. 4A) or in the average mark for the short answer component of assessments (Fig. 4B). We then sought to determine whether blended learning had made any changes in students’ learning in the three learning categories of knowledge and understanding, communication and application, and thinking and inquiry. We observed that there were no significant differences in the proportion of students who obtained a correct response to multiple‐choice questions in each of these learning categories with the implementation of blended learning (Fig. 4C). There was also no significant difference in the average within‐semester change in short answer marks between term tests 1 and 2 when comparing both before, and after the implementation of blended learning in the BIO1A03 course (Fig. 4D).

### RQ2: Student perceptions of blended learning in the BIO1A03 course and impact on their scientific learning

**Fig. 5:**
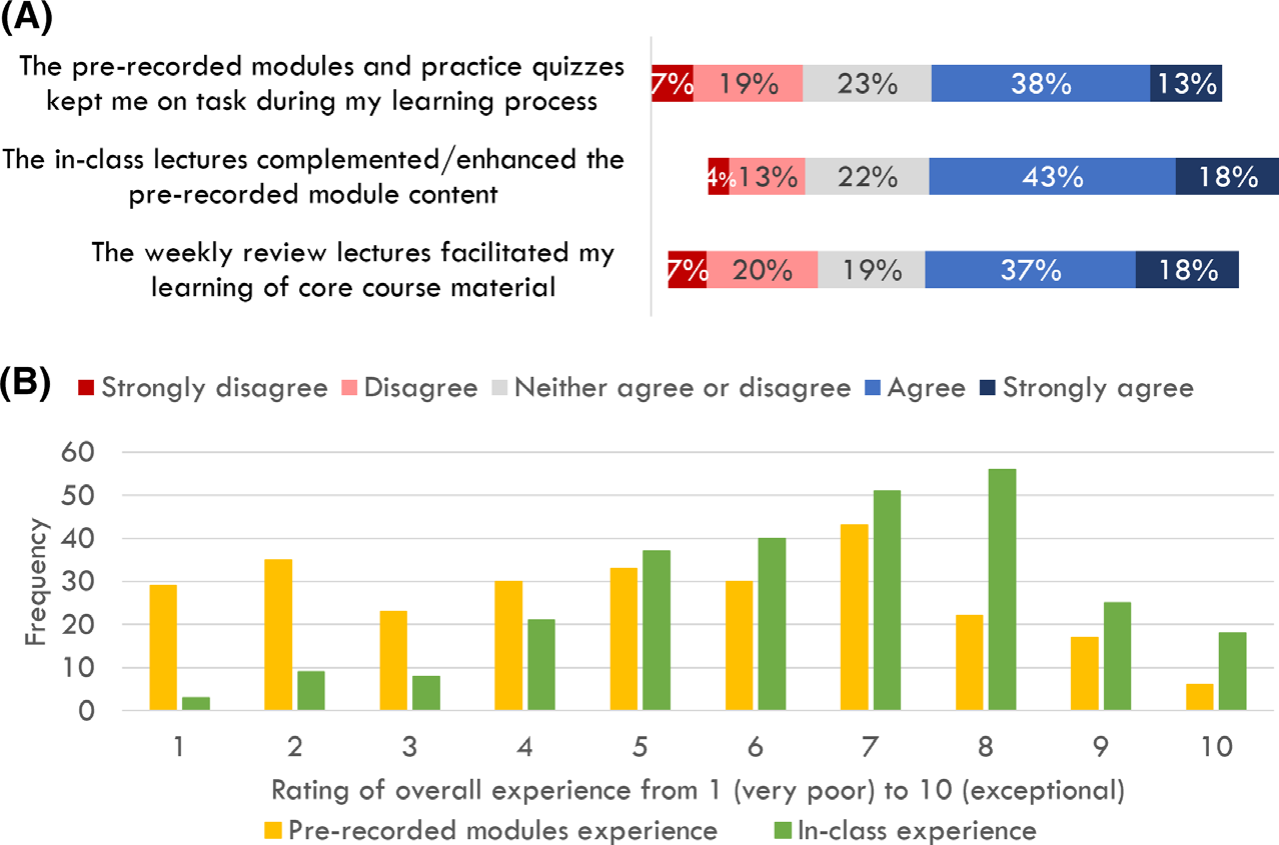
Student responses regarding the blended learning format of the BIO1A03 cellular and molecular biology course at McMaster University. These include responses related to (A) survey questions assessing different blended learning components (*N* = 271) and (B) ratings of their overall student experience with the prerecorded modules and in‐class lectures (*N* = 268).

Of the 1593 students enrolled over the fall 2017 and winter 2018 semesters of BIO1A03, 282 (17.7%) completed the survey and 24 students (1.5% of the cohort) participated in the focus groups, with 10 students participating in one‐on‐one interviews, and 14 participating in one of four focus groups (ranging from two to five students per focus group). Of the 24 focus group participants, 19 (79%) had also completed the survey. Interviews and focus groups ranged in duration from 20 to 80 min (with an average duration of approximately 30 min each). The coded themes and subthemes that arose from the sentiment analysis are illustrated in Table 1; examples of coded quotes are described in the text below. In general, students appeared to agree that the three components of the blended learning format (i.e., the online modules, in‐class lectures, and review lectures) supported their learning (Fig. 5A). This was corroborated by what students had to say during the interviews. Students described ‘really lik[ing] the modules because [they] can go back anytime and write notes again’. They emphasized that ‘this was the only class that [they were] taking that has an applied lecture component and [that they] found it really interesting and useful…[that] it kind of made [the module content] relevant to the world’. Students appreciated the review lectures in that ‘if you still don’t get something [after doing the modules], you can just take some time off and go to review lecture’. However, it was quite clear that students perceived their in‐class experience (including both review and applied lectures) as better than their web‐module experience (median_Web modules_ = 5 out of 10, median_In‐class_ = 7 out of 10, χ_2_ *P* < 0.005) (Fig. 5B).

**Table 1:** Coded themes and subthemes for student responses to focus group questions (*n* = 24).

Theme	Subtheme	Number of unique responses coded to each subtheme (% total)
Knowledge and understanding	Modules improve retention of biology content	45 (4.3)
	BL format improves understanding of content	27 (2.6)
	Volume of content delivered in modules impaired student understanding	24 (2.3)
	No impact of BL on knowledge and understanding	4 (0.4)
	Students’ expectations of breadth and depth of course	50 (4.8)
Communication	Improves communication of biology content with others within and outside the course	38 (3.6)
	Worsens ability to communicate biology content within and outside the course	41 (3.9)
	No impact of BL on communication	29 (2.8)
Application	Improves application of content to real‐world setting	13 (1.2)
	Decreases ability to illustrate applications	3 (0.3)
	No impact of BL on application	2 (0.2)
Critical thinking and inquiry	BL provided multiple opportunities draw connections between content	24 (2.3)
	BL has other benefits for critical thinking and inquiry	2 (0.2)
	No impact of BL on critical thinking and inquiry	9 (0.9)
Impact of BL on studying habits and academic activities	Viewing content	79 (7.5)
	Studying	47 (4.5)
	Reviewing	9 (0.9)
	Practicing	3 (0.3)
	Assessment	13 (1.2)
Hopes for BL in future courses		14 (1.3)
Perceptions of impact of BL on success in BIO1A03	Positive	10 (1)
	Negative	1 (0.1)
	No impact	2 (0.2)
Emotions related to blended learning	Positive (joy, satisfaction, etc.)	15 (1.4)
	Negative (fear, worry, anxiety, etc.)	20 (1.9)
	Ambivalence	8 (0.8)
Suggestions for improvement of BL components	Online modules	144 (13.8)
	In‐person components	48 (4.6)
	Formative and summative assessments	44 (4.2)
Time management	BL allows for better time management	40 (3.8)
	BL impairs time management	50 (4.8)
Workload	Compared with other BL courses	31 (3.0)
	Compared with nonblended learning courses	35 (3.3)
Advantages (not related to other themes)		40 (3.8)
Disadvantages (not related to other themes)		28 (2.7)
Responses not related to above themes		100 (9.6)

**Fig. 6:**
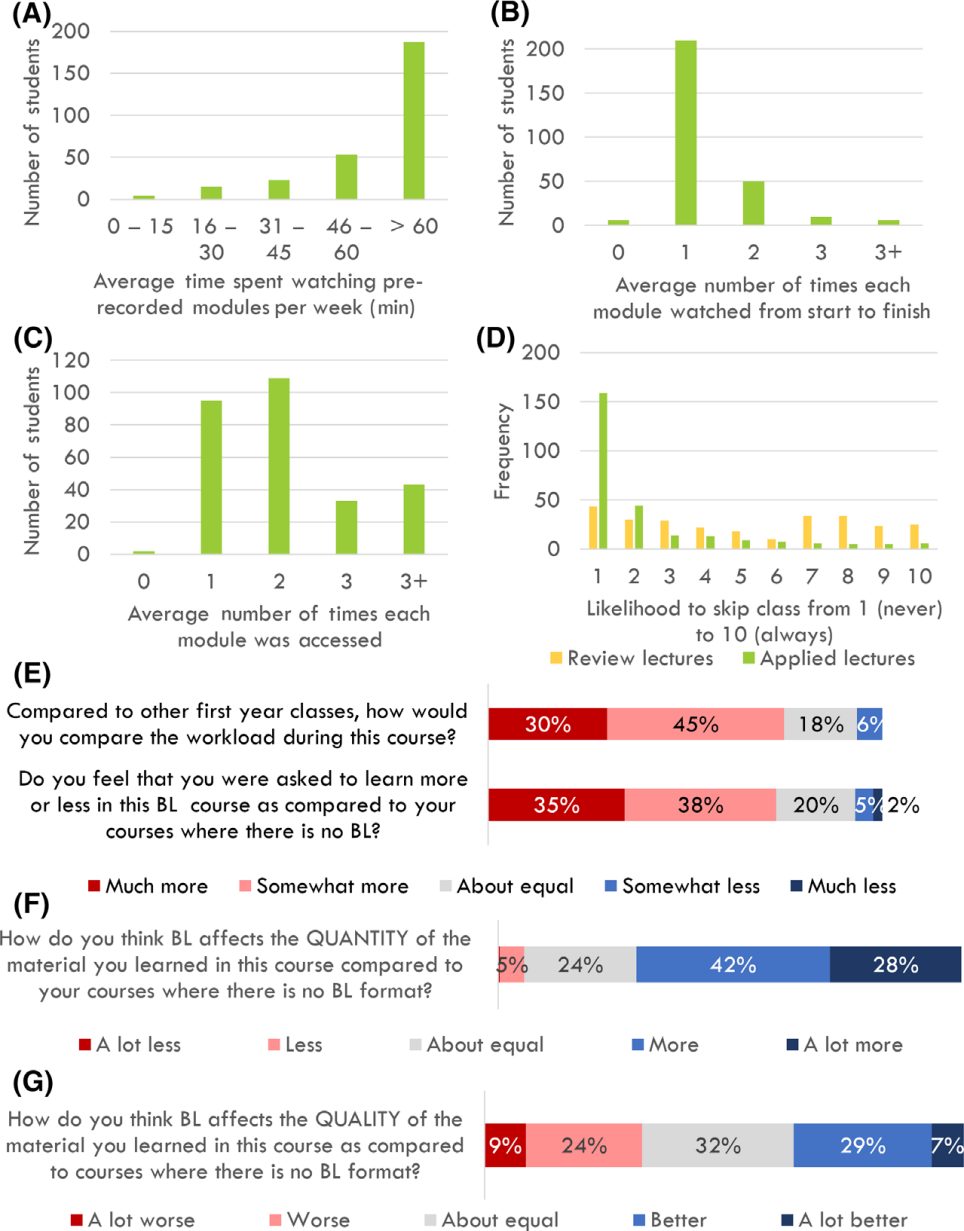
Student responses regarding (A) time spent on each prerecorded module, (B) number of times each module was watched, (C) number of times they accessed a module, (D) how likely they were to skip review and applied lectures, (E) perceived workload, and how they felt the blended learning format of the BIO1A03 cellular and molecular biology course at McMaster University affected the quantity (F) and quality (G) of material that was learned when compared to courses where there is no BL format (*N* = 282).

Further data obtained provided insight into why the prerecorded modules were not rated as highly as the in‐person review and applied lectures. Specifically, we found that despite most of the web modules being 25–40 min in duration, the majority of students spent more than 60 min on each module (Fig. 6A). Students indicated that ‘it [would] take at least twice the amount of time to get through the module as [compared to] the time length [indicated on the learning management system]’. Several students suggested that the length of time that was required to dedicate to the modules substantially increased their workload, to the point that they felt that ‘biology was taking up [the] entire week’. This perhaps also explains why many students felt that the workload in BIO1A03 was higher compared with their other first‐year classes (including other courses with a blended learning format), and why they felt that they were being asked to learn more in this course compared with their traditionally formatted courses (Fig. 6E). One student suggested that another factor contributing to this perception of a higher workload was a sense of discontinuity between the various components of our blended learning approach:

[I]t felt like there was a lot because…you were watching the modules, and then also attending [review] lectures, and then there were also the applied lectures, so it felt like there were three different kinds of sections to learn.

Despite these perceived challenges, the majority of students responded that they learned more or a lot more due to the blended learning format (Fig. 6F). The data suggest there is almost no agreement concerning the impact of blended learning on the quality of the material they learned (36% thought it was better while 33% thought it was worse) (Fig. 6G). Students suggested that:

Since it was blended learning, they were able to incorporate all the information and go in‐depth with the modules. If it wasn’t, if it was just in‐classroom, I don’t [think] the [professors] would have been able to teach us all of that.

Several students suggested a number of benefits to the blended learning format during the focus groups. A significant proportion of the benefits were related to the web modules. Major benefits that emerged from the focus group discussions related to increased flexibility and improved time management. For example, the majority of students cited that the web modules ‘allow for a lot of flexibility between other classes…I can work on it at my own time’. This increased flexibility can also be inferred from the survey results that reported that, despite the majority of students watching each module from start to finish only once (Fig. 6B), more than half accessed each module two or more times (suggesting that they were breaking up the modules and watching them at their own pace) (Fig. 6C). The second major benefit that students discussed was the fact that the web modules provided a consistent resource that they could return to, either because they needed to review a difficult concept several times or because they needed to review material for an assessment. For example, one student mentioned that ‘[If] I didn't fully understand a concept in lecture, I might forget to go over it after the lecture. Whereas in the web modules I'll realize "Oh, I didn't get that" and I'll just go over it again until I get it’. They also highlighted this by setting the BIO1A03 course in context to their other courses. For example, one student mentioned that with other non‐BL courses, ‘…[the content is] said in‐class once and that’s it. It’s harder to go back and re‐teach yourself things when you don’t have a reference’.

Students also emphasized the advantages of the applied lectures. As previously noted, the majority cited how the applied lectures made content ‘relevant to the world’. They also noted that it was some of the ‘most interesting material’ that they were learning and made them ‘excited about biology’. Interestingly, several students themselves noted that by partitioning the material into core content delivered through the modules followed by applied lectures, the course increased their learning because it allowed for ‘repeated exposure’ to ‘build on what [they were] learning’, and to go more in‐depth because the instructors ‘don’t have to spend the whole lecture time explaining all the little steps’. For example, a student indicated that:

[The blended learning format] kind of separated the concepts from the applications because we were taught it at different times. Like, in the web modules [there are] the concepts, and then in the applied lecture we learned the applications of it…. It allowed us to learn everything and get a good understanding of it and then learn the applications, instead of having applications placed throughout the lecture as we were learning the concepts…It was good!

Students were particularly positive about the format of having review lectures after the modules because ‘[they] found that when [they were] asking questions on the day of the [review] lecture, by viewing the module and having key terms written down, [they were] able to ask more effective questions because [they] knew the terminology’. However, while considering these perspectives, it is important to note that when asked to rate how likely they were to skip the review and applied lectures from one (never) to ten (always), students were much more likely to skip review lectures (median_Review_lectures_ = 5) compared with applied lectures (median_Applied_lectures_ = 1) (χ_2_ *P* < 0.005) (Fig. 6D).

### Perceptions about the impact of blended learning on the learning categories

**Fig. 7:**
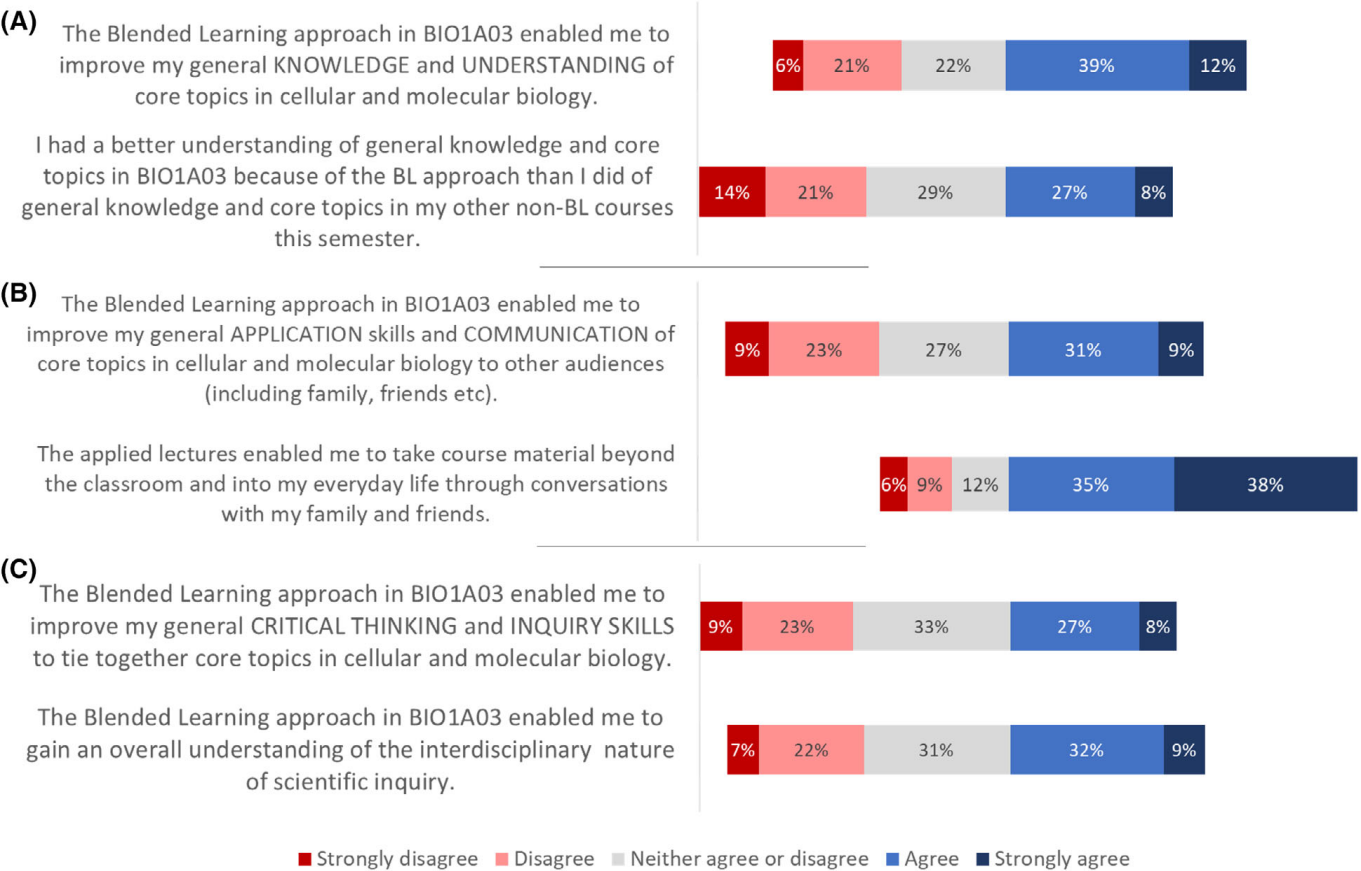
Student responses related to their perceptions regarding the impact of blended learning on their scientific literacy in the areas of (A) knowledge and understanding (KU), (B) communication and application (CA), and (C) thinking and inquiry (TI), (*N* = 282) in the BIO1A03 cellular and molecular biology course.

Despite not observing any changes in student performance in the three learning categories after the implementation of blended learning, we wanted to understand whether students perceived any differences in their knowledge and understanding (KU), communication and application (CA), and thinking and inquiry skills (TI) in this BL course, as compared to their experiences in other courses. While the majority of surveyed students felt that the blended learning approach enabled them to improve their general knowledge and understanding of core topics in cellular and molecular biology, students were approximately equally split as to whether they had a better understanding of these topics in this course as compared to their nonblended learning courses (Fig. 7A). When asked during the focus groups and interviews about how students felt BL influenced their general knowledge and understanding of core topics in cellular and molecular biology, students were similarly divided. They reported that one major advantage to the blended learning format was that since the modules allowed the course to ‘pack tons of information, they were forced to learn more’. However, this same quantity of information was a disadvantage for other students, who felt that the ‘content was thrown at [them]’, which may have ‘hindered their knowledge’, and thereby decreased their understanding. It is important to note, however, that the foundational curriculum had not changed before and after the implementation of blended learning. Indeed several students noted that ‘I think the biology course just holds that much content….I don’t know if it has to do with blended or not’ and regardless of whether it was blended learning or traditional [they] would have still been learning the same amount of content. Therefore, it is unclear whether some students’ negative perceptions about the impact of a large amount of content on knowledge and understanding are due to the BL format of the course or to the nature of biology as a content‐heavy discipline. Finally, the majority of students emphasized the flexibility that blended learning offered, mentioning that ‘it allowed [them] to take [their] own time to actually understand the concepts, instead of having to learn it [...] quickly when they are teaching it in class’ and that this flexibility was essential in allowing them to solidify key concepts.

Regarding communication and application, survey responses indicated that slightly more students agreed or were neutral to the statement that the BL course format enabled them to improve their general application and communications skills, especially with other audiences (Fig. 7B). A key factor that seemed to play into this increased ability to communicate with people was that students in focus groups felt the blended learning format as a whole was conducive to having discussions both in‐class and online. Students mentioned that, since they were watching the modules outside of scheduled class time, they were able to ‘watch the modules together with friends and to discuss any uncertainties with them more frequently’ increasing their ability to communicate with others. Students also felt that the modules increased their opportunities to engage in biology‐based discussions outside of their courses. For example, one student indicated:

I think like when I am watching a module at home or at a library with [my] friends, there are people around you, so if [I] hear something interesting, it’s more likely that [I] will kind of pause it there, and [say] “did you know for example that… baby’s hemoglobin have more affinity towards oxygen than the mother’s hemoglobin?”…But if I am in the lectures, I’m not going to be like, “Stop!”, just to tell people.

Moreover, when asked on the survey whether the applied lectures enabled them to take course material beyond their classroom and into everyday life through conversation with family and friends, more than 74% agreed or strongly agreed. This indicates that students perceived the applied lecture components as being particularly important when it came to applying content beyond the classroom and to communicate with others (Fig. 7B). For instance, several students responded with comments such as:

I just enjoyed [the applied lectures] because it showed you what you could actually do with what you're learning and how what you're learning applies to the real world. Sometimes I feel like, in chemistry or something, I feel like I'm learning things, but I don't know how it applies to the real world.

I keep coming back to it… so it must be that the applied lectures really made me even more interested in the content. It made it less abstract, the things we were learning, which I think is really important for something like biology. So, I was already interested in these things coming into the course, but I definitely enjoyed it. I love the applied lectures.

It is quite clear that students indicated great satisfaction with the applied lectures and for giving them a better understanding about the application of the content they were learning. Regarding communication, the majority of students indicated that ‘with the applied lectures [they] understood the main ideas a lot better; and so, [they] would more effectively be able to communicate that with other people’.

Most students reported only slightly positive perceptions of the blended learning approach and whether it enabled them to improve their general critical thinking and inquiry skills in biology or whether the blended learning approach enabled students to gain an overall understanding of the interdisciplinary nature of scientific inquiry (Fig. 7C). In fact, many of the students seemed to be neutral about the impact of blended learning on their thinking and inquiry skills. This was also revealed in our discussions with students who indicated that the major benefit to the blended learning format was that it increased their opportunity to draw connections at their own pace—thereby increasing their critical thinking. For example, responding to a question about the impact of blended learning on critical thinking and inquiry, one student mentioned that:

When you are watching a module, you kind of have more time to process information so…as I’m watching it, just because I have more time to think about it, I [am] able to relate it to something else I’ve learned before. [In comparison], during a lecture it’s kind of more stressful because the instructor only has 50 minutes, and it’s going really quick so you kind of just take notes. So, I don’t think at that moment [I’m] making the connections…

However, other than a few comments related to critical thinking and inquiry, during focus groups and interviews, approximately half of the participants indicated that they ‘didn’t think it really impacted [their] critical thinking or inquiry’.

### Student suggestions for improvement of the blended learning course

Although our primary objective was to understand student perceptions regarding the BL format more generally and its impact on student performance in the three learning categories, more than 20% of the references we coded from our transcripts were related to potential improvements to the blended learning format. Firstly, students felt that the modules were currently too long and, that they sometimes ‘zoned out’ while listening. To address this, they suggested that the modules ought to be more ‘concise’ and ‘succinct’. Other students indicated that they felt that the modules should be divided into much shorter videos. A second major point was that students indicated they felt the modules were ‘monotone’ or ‘boring’. Thirdly, many students indicated that this course was, for many of them, their first exposure to a BL formatted course where modules were being used to deliver the majority of the content. They felt that they hadn’t been sufficiently instructed by the course instructional team on how to best utilize the modules, have proper time management, and balance the various learning resources available. They felt left alone to learn how to navigate the course themselves—leaving them to flounder, which resulted in struggles during the first several weeks of the course. Accordingly, several students suggested that the course instructors should take a more active role in guiding the students in how to make the best use of their various resources (via an announcement, an introductory video, etc.). Lastly, students indicated that the modules ought to have increased opportunities for self‐assessment, such as through increased ‘checkpoints within the modules’ and exit quizzes. This was despite the existing modules already having learning checkpoint questions. Students felt that they needed an increased number of questions and more ‘difficult’ questions to better gauge their understanding of the material.

## Discussion

Through this study, we have examined the impact of BL on student performance and perceptions of learning in an introductory cellular and molecular biology course. We examined the impact of the BL learning approach on students’ performance in multiple‐choice and short answer questions and found no differences in student performance. Our results are aligned with the findings of several other studies [8, 30, 31], which also reported no statistical difference in student performance following the implementation of blended or hybrid teaching approaches in courses. Early work evaluating the relative efficacy of different teaching modalities has shown that differences in student performance may depend on the level or order of learning outcomes, with online and in‐person delivery methods being more favorable for lower order thinking skill development, but in‐person instruction being preferable for the development of higher order thinking skills [32]. In contrast, our retrospective study has shown that there are no statistically significant differences in student performance at all orders of learning (knowledge and understanding, communication and application, and thinking and inquiry) when comparing pre‐ and postblended learning models of the BIO1A03 course. Conversely, other case studies have suggested that blended learning approaches can effectively be used to improve student performance in HOTS [33]. Similar to our applied lectures, dos Santos Czepula et al. contextualized their course content with the aim of giving meaning to learning. However, unlike in our study where the Bloom’s taxonomy was used to categorize questions *post hoc*, dos Santos Czepula et al. developed their assessment questions to include all levels of Bloom’s taxonomy *a priori*. This suggests that an intentional *a priori* approach to developing assessment questions that capture all levels of Bloom’s taxonomy may be the key to improving student performance in a blended learning setting. Interestingly this might also be implied from student comments that questions in formative assessments would benefit from being more challenging.

Contrary to our hypothesis, students entering the post blended learning course did not perform worse on assessments compared with students completing the preblended learning course. This was despite focus group participants noting, as we expected, a relative unfamiliarity with the blended learning approach and a desire for more guidance on how to maximize the efficiency of their learning. Similarly, unlike our assumption that students increased familiarity with the blended learning approach would lead to a concomitant increase in student performance during the second term test in the BL version of the course, we found instead that there was a nonsignificant trend towards students achieving more poorly in the second test (Fig. 4D). This trend is challenging to explain since it depends on a number of variables outside the control of the biology team. For instance, several focus group participants commented on the impact of the course loads of other blended learning courses at our institution and how course load in general tended to get heavier as the semester progressed. They also commented on assessments being too close to one another. While this is not an issue isolated to blended learning approaches, it does imply the need for better coordination between course administrators to ensure that students are not overwhelmed and have an optimal learning experience. However, we can expect that future cohorts of students will be more familiar with online and blended learning pedagogy due to the impact of the COVID‐19 pandemic.

It was interesting to note that while most students perceived that they learned more or a lot more due to the blended learning format, students were equivocal about the impact of blended learning on the quality of their learning. This could be explained by the fact that while students likely have similar definitions of ‘quantity’, ‘quality’ of learning is a more ambiguous concept, and therefore, students likely interpreted our survey question in different ways. To gain more insight into these student perceptions, it could have been more valuable to ask specific questions using existing frameworks about education quality [34].

Students spoke about several benefits of the BL format of the BIO1A03 course. A large proportion of these benefits are centered around self‐paced learning and flexibility [35]. However, the flexibility of the course may also be a disadvantage for some—for instance, although the review lectures were not mandatory and may not be useful for excelling students, students were more likely to skip review lectures despite it being an important part of our course framework and an opportunity to clarify poorly understood concepts. These findings are not novel and closely reflect previous findings by other authors [36, 37, 38]. However, students' stated needs for better time management must be interpreted cautiously. The majority of study participants were first‐year students entering university, and their comments may not necessarily reflect the blended learning approach in this course but rather the acclimatization to university education. This aligns with previous studies, which have shown that student success is greatly related not only to self‐motivation but also to time management [39]. Another benefit students appreciated was that, since foundational content was delivered via the online modules, in‐class time could be used to facilitate the application of principles, which led to consolidation [13]. They also reinforced a stronger appreciation for real‐world applications of biology. Several students commented on how applied lectures also spot‐lighted potential job opportunities.

The study has also provided various recommendations and suggestions for the improvement of components of our blended learning approach. A number of recommendations were centered around the online modules. Students noted that modules were taking significantly longer to complete and suggested reorganizing the modules into smaller subunits. This aligns with our data, which show that while students would only watch the entire module once, they were accessing the video more than once in order to break the module down into smaller chunks. This suggestion aligns with the current literature on cognitive load theory. Cognitive load theory defines ‘learning’ as the development and automation of cognitive schemas stored in long‐term memory. Studies suggest that, to respect the limitations of human working memory, there is a need to minimize cognitive load. One recommendation is to design learning sources to minimize the working memory needed for cognitive processes that do not contribute to learning, by for instance presenting information in the correct manner (e.g., visually) and presenting information in a single source [40, 41]. It also involves presenting information in digestible chunks. Evidence supporting this theory includes a study by Humphries and Clark who illustrated that providing didactic information in small chunks (3‐ or 17‐min videos vs. one‐hour didactic lectures) was associated with stronger student preference, increased cumulative use, increased completion of instructional material, and improved student grades [42]. Based on findings such as these, current guidelines on cognition in education indicate that six to nine minutes is the optimal length for a video in order to sustain audience attention [43]. This implies that having better approximations of completion time can help students facilitate better time management when reviewing and studying the prerecorded modules. Students have also suggested that the module can be improved to be more interactive and engaging. This sheds light on the need to revise the modules regularly to include interactive components such as virtual labs and experiments that students can work through to facilitate further understanding of the core course material.

Another recommendation was to provide more frequent testing opportunities as a means for students to self‐appraise their learning. It is well recognized that formative performance feedback is a valuable tool in the learning process, especially when it provides students the opportunity to gain insight into their personal understanding of course content [44]. Despite the fact that the prerecorded modules already provide existing checkpoint questions, the majority of students felt that existing questions were too easy and that they did not reflect the types of questions seen in assessments. Currently, the majority of these module checkpoint questions would fall under the knowledge and understanding category of questions. As others have noted [45], the quizzing of lower order thinking skills does not translate to improved performance on higher order thinking skills. This could explain why students felt that current questions were insufficient and suggest that questions assessing conceptual understanding, application, and critical thinking should be added to the module checkpoints to improve their ability to provide formative feedback to students.

During program development, the blended learning curriculum team intended to create a seamless course with clear associations between foundational principles taught in weekly modules and the weekly applied lectures. Consequently, it was surprising that some students described a sense of discontinuity between these two aspects of our blended learning approach or felt that they were separate sections to learn. On the other hand, as previously noted, the majority of students provided a clear appreciation for the differentiation of module and applied content, stating that it allowed for both the consolidation of foundational content before its application and that the repetition created better consolidation.

### Limitations

Our study had several unavoidable limitations. Firstly, with regard to our quantitative analysis of student performance, the course only provided anonymized data on the proportion of students that had correctly answered a multiple‐choice question without information linking multiple‐choice answers to a particular student. Accordingly, it was impossible to determine patterns at an individual student level. Additionally, when it came to certain statistical analyses, we were limited by our number of data points. For example, after the implementation of the BL course format, only one exam contained thinking and inquiry multiple‐choice questions, compared to four exams with thinking and inquiry questions in the traditional approach. This potentially limited the power of that specific statistical analysis [46]. This also limits our ability to make conclusions on the impact of blended learning on student performance in thinking and inquiry HOTS. Another limitation is that we were unable to capture demographic data about focus groups and interview participants. While we obtained a robust sample size, we cannot be certain whether their perspectives provide a generalizable representation of the entire class. For instance, it could be that focus group participants consisted only of students with poor marks and therefore poor perspectives of the course. Moreover, because of significant limitations in student scheduling and availability, some participants were interviewed in focus groups while others were interviewed in individual interviews. This approach, while necessary, differed from our a priori intention to complete focus groups only. However, using both research techniques may have allowed us to resolve each technique's disadvantages and to generate a richer understanding of student perceptions [47]. As noted by Kaplowitz and Hoehn [48], focus groups rely on group dynamics to reveal participants' similarities and differences of opinion and are therefore subject to group effects. These effects may facilitate an exchange of ideas and stimulate individual group members’ thinking, allowing group members to build on each other’s ideas. On the other hand, group members might not exchange all the information they have, might focus only on shared information, or problems related to dominant group members and social pressure may lead to incomplete or biased information processing. These are all issues out of the research team’s control and are a part of any study of this nature. Contrastingly, students in individual interviews may have been more candid, and we were able to go significantly more in‐depth into their experiences. Finally, students’ perceptions of BL may have been impacted due to their acclimatizing to university and undergraduate studies. Our study did not ask students about how that transitional process factored into their perception of BL and its impact on their learning and performance.

### Future directions

As a research team, we have completed a mixed‐methods analysis of the impact of a first‐year cellular and molecular biology course with a BL format on student performance in the learning categories of knowledge and understanding, communication and application, and critical thinking and inquiry. We have also illustrated that students have mixed sentiments about the BL format and its impact on their learning. While we report no statistically significant difference in student performance in these learning categories, our research is in line with current Commonwealth of Learning guidelines, which emphasize that there should be no quality difference between online and in‐person classroom instruction in achieving student performance [49]. We are interested in evaluating whether a BL course format may be more impactful on performance as students progress through higher postsecondary levels. At a time when much of the global student population has already been displaced from the in‐person classroom due to the COVID‐19 pandemic, it will also be interesting to evaluate these learning outcomes and student perceptions of BL with cohorts of students who enter university with a stronger exposure to BL based on their introduction to this instructional format while in secondary school. From a long‐term perspective, most students reported an increase in their ability to apply course concepts to the real world. Given this, it will be valuable to track student performance beyond their first year and compare the performance of students who completed the BIO1A03 courses in a BL format, with that of students who completed the course in a traditional format. Finally, challenges students incur in transitioning between secondary and postsecondary environments and the impact that BL has on this process should be further investigated to better ease that transition in future BL analyses. Since anonymization of student marks and performance was a requisite for ethics approval at our institution, we were unfortunately unable to link student sentiments and demographic information with their individual performance. However, a future prospective study direction would be to assess how student perceptions and demographic information (such as their academic preparation, socioeconomic status, and career goals) modify the relationship between blended learning and student performance. Similarly, future studies will also aim to investigate how students’ perceptions about blended learning impact student learning behaviors.

### Insights into best practices in blended learning

Abstracting from our study findings, we present several general insights into best practices that can be implemented in both local and global blended learning settings:

1. Instructors must be cognisant that a student's workload should not increase when transitioning a course to a blended learning format.
2. Students should be introduced to the blended learning method and provided instructions on how to efficiently use the course content.
3. Prerecorded video content provided in a blended learning format should follow current principles of cognition in education, which suggest an optimal video length of six to nine minutes. This will maximize learner flexibility and is more likely to optimize learning.
4. When using formative checkpoints within educational content delivered through online platforms, checkpoints should appraise lower and higher order thinking skills and should be reflective of the level of difficulty of questions in summative assessments.
5. Prerecorded video content should maximize the opportunity for students to integrate and apply course content to the ‘real world’, either through subsequent synchronous or asynchronous applied lectures.

## Conclusions

As educators, we appreciate that students find great benefit when ample time is specifically allocated towards applying course content to the real world. As we emerge from this monumental pandemic, we must all reflect on not only what we teach, but why and how, with the goal of preparing our students to be problem‐solvers of the future, who can be motivated to solve emerging and urgent global challenges [14]. As we reported, the BL format of the BIO1A03 course affords the instructional team with this opportunity, without compromising student performance. The remodeling of core course content into prerecorded online modules, and the opportunity to review this material with course instructors during a separate lecture, provides the instructional team with a full lecture that can be dedicated towards the meaningful application of course material to the real world. Throughout the COVID‐19 pandemic, this current BL course model facilitated increased learning flexibility (which has become so important throughout the last two years). The BL format also allowed us to incorporate relevant just‐in‐time real‐world applications of biology related to the pandemic (such as learning how viruses are able to highjack cellular machinery). The data obtained from this study provided us with confidence that our BL model would be impactful during this disruptive period. We encourage all instructors who are inclined towards blended learning to consider a shift in this direction, while keeping the insights into best practices in blended learning in mind.

## Conflict of interest

Rosa da Silva and Alastair Tracey were part of the BIO1A03 blended learning instructional team. As such, all data analysis and interpretation were carried out by all other study authors. All remaining authors have no other conflicts of interest.

## Author contributions

RDS, AT, IT, and WY conceived and designed the project. IT and VR acquired the data. IT, VR, and VV analysed and interpreted the data. IT, RDS, and VV wrote the paper.

## Supporting information


**Data S1**. Survey and interview questions utilized to evaluate human and social impacts of blended learning on student perceptions. These questions were utilized through both online surveys and in‐person focus groups respectively.

## Data Availability

Research data are not shared due to student privacy restrictions.
