# Supplementary material for: Blended learning in a biology classroom: Pre‐pandemic insights for post‐pandemic instructional strategies
Source: FEBS Open Bio. 2022 May 23;12(7):1286–305. doi: 10.1002/2211-5463.13421 (PMC9249331; doi:10.1002/2211-5463.13421)
Supplement: Supplementary file 1 — Data S1. Survey and interview questions utilized to evaluate human and social impacts of blended learning on student perceptions. These questions were utilized through both online surveys and in‐person focus groups respectively. [file FEB4-12-1286-s001.docx]

## Supplemental Material

Survey Questions:

1. In which faculty are you enrolled?
2. Why did you take the course?
3. On average, how much time did you spend on each web module?
4. How many times did you watch each module from start to finish?
5. How many times did you access each module?
6. The web module checkpoint questions engaged my attention and enhanced my learning experience.
7. The web module theme quizzes enhanced my learning, and helped prepare me for the evaluations.
8. The online modules and practice quizzes kept me on task during my learning process.
9. The in-class lectures complemented/enhanced the web modules content.
10. The weekly review lectures facilitated my learning of core course material that was delivered via online modules.
11. The applied lectures enabled me to take course material beyond the classroom and into my everyday life through conversations with my family and friends.
12. This course increased your interest in biology.
13. Are you now considering taking futures courses in biology?
14. Compared to other first year classes, how would you compare the workload during this course?
15. Please rate your web modules experience on a scale of 0 - 10 (0 being very poor, 10 being exceptional).
16. Please rate your in-class experience on a scale of 0 - 10 (0 being very poor, 10 being exceptional).
17. On a scale of 1 to 5, how would you rate your lab skills in biology, BEFORE you started university? (1 being skills need development, 5 being excellent)
18. The lab content complemented/enhanced the content presented during the in-class lectures and the web modules.
19. The main laboratory big-research question (Amylase gene copy number variation in populations) motivated me to study course material
20. The procedures in the lab manuals were clear and easy to follow.
21. Please rate your lab experience on a scale of 0 - 10 (0 being very poor, 10 being exceptional).
22. On a scale of 1 to 5, how would you rate your lab skills in biology after completing Biology 1A03? (1 being skills need development, 5 being excellent)
23. The Blended Learning approach in BIO1A03 (combining online learning with face-to-face in-class and laboratory instruction) enabled me to improve my general knowledge and understanding of core topics in cellular and molecular biology.
24. The Blended Learning approach in BIO1A03 (combining online learning with face-to-face in-class and laboratory instruction) enabled me to improve my general communication skills and application of core topics in cellular and molecular biology to other audiences (including family, friends, etc.).
25. As the BIO1A03 Blended Learning Course progressed throughout the semester, I felt more comfortable asking questions in class or contributing to class discussions.
26. The Blended Learning approach in BIO1A03 (combining online learning with face-to-face in-class and laboratory instruction) enabled me to improve my general critical thinking and inquiry skills relative to tying together core topics in cellular and molecular biology.
27. The Blended Learning approach in BIO1A03 (combining online learning with face-to-face in-class and laboratory instruction) enabled me to gain an overall understanding of the interdisciplinary nature of scientific inquiry.
28. I felt more prepared for tests and the final exam in BIO1A03 because of the BIO1A03 Blended Learning approach than I did for my non-blended learning courses this semester.
29. I had a better understanding of the BIO1A03 general knowledge and core topics because of the BIO1A03 Blended Learning approach than I did of general knowledge and core topics in my other non-blended learning courses this semester.
30. My final mark in Grade 12 biology was:

Interview Questions:

1. How many courses are you now taking that are in a Blended Learning format?
2. How do you feel the workload compares in BIO1A03 with these other courses?
3. Do you feel that you were asked to learn more in this blended learning format course as compared to your courses where there is no blended learning format?
4. Do you find that Blended Learning helps to manage studying schedule more effectively?
5. Are there any drawbacks with Blended Learning?
6. Would you change the Blended Learning format of the BIO1A03 course?
7. Do you hope that future courses incorporate Blended Learning as part of their course instruction model?
8. Do you think that Blended Learning positively impacted your success in the course?
9. How do you feel Blended Learning influenced your general knowledge and understanding of core topics in cellular and molecular biology?
10. Was the depth and breadth of what you learned consistent with your expectations? Do you think that this had anything to do with the blended learning format?
11. How do you think blended learning affects the quantity and quality of the material you learned in this course? Do you think you learned more or less? Do you think you retained more or less?
12. How do you feel Blended Learning influenced your general communication skills, asking of questions, engagement in discussions and application of core topics in cellular and molecular biology to other audiences (including family, friends, etc.)?
13. How do you feel Blended Learning influenced your general critical thinking and inquiry skills relative to tying together of core topics in cellular and molecular biology?
14. How do you feel that the blended learning format affected your opportunities to discuss questions and understand material with your professors/classmates/TAs in comparison with a classically taught course?
15. What do you think prevented you from performing better during the midterms? How was your experience with the types of questions? Did the type of material you learned through class align with your experience with the midterm questions?
